# Combined extracts of *Garcinia mangostana* fruit rind and *Cinnamomum tamala* leaf supplementation enhances muscle strength and endurance in resistance trained males

**DOI:** 10.1186/s12970-018-0257-4

**Published:** 2018-10-22

**Authors:** Manikyeswara Rao Konda, Krishnaraju Venkata Alluri, Prason Kumar Janardhanan, Golakoti Trimurtulu, Krishanu Sengupta

**Affiliations:** 1 Suraksha Health Village, 121, SBH complex, Gurunanak Nagar Road, Vijayawada, Andhra Pradesh India; 2 Laila Nutraceuticals R&D Center, 181/2, JRD Tata Industrial Estate, Kanur, Vijayawada, Andhra Pradesh 520007 India

**Keywords:** *Cinnamomum tamala*, Endurance of exercise, Forced swim test, Free testosterone, *Garcinia mangostana*, Lean body mass, Muscle strength

## Abstract

**Background:**

A proprietary composition GMCT contains extracts of two popular Asian herbs viz., *Garcinia mangostana* (GM) fruit rind and *Cinnamomum tamala* (CT) leaf. We systematically evaluated physical performance and muscle strength enhancing ability of GMCT in a preclinical mouse model followed by a 42-days double-blind placebo controlled human trial in resistance trained adult males.

**Methods:**

Four groups of Swiss albino mice (20–30 g body weight) (*n* = 6) were fed a standard laboratory diet and given Carboxymethylcellulose sodium (CMC), 150 mg/kg GMCT (GMCT-150), 300 mg/kg GMCT (GMCT-300) or 50 mg/kg Oxymetholone (OXY) via oral gavage for 21 days. On day 22, the animals’ physical performance and muscle strength were assessed in a forced swimming test (FST) and forelimb grip strength experiment, respectively.

In the human trial, thirty-eight resistance-trained young adults (mean age 26.32 ± 4.39 years, body weight 67.79 ± 12.84 kg, BMI 22.92 ± 3.54 kg/m^2^) completed the trial. The participants received either GMCT (*n* = 19; 800 mg daily) or matched placebo (n = 19) for 42 days. As primary variables, 1-RM bench press, 1-RM leg press, and leg extension repetitions were measured at baseline and on days 14, 28 and 42 of the intervention. Anthropometric parameters and serum markers such as free testosterone, insulin-like growth factor 1 (IGF-1), insulin and lactate were also measured before and after the intervention.

**Results:**

GMCT-300 mice showed significant improvement in swimming time (GMCT: 395.3 ± 81.70 s vs. CMC: 271.6 ± 56.86 s; *p* = 0.0166), distance (GMCT: 341.22 ± 65.88 m vs. CMC: 260.84 ± 49.15 m; *p* = 0.0461) and grip strength (GMCT: 43.92 ± 6.97 N vs. CMC: 35.0 ± 6.92 N; *p* = 0.0490), compared with the CMC group.

At the end of the 42-day human trial, the per protocol analyses reveal that mean changes from baseline 1-RM bench press (GMCT: 23.47 ± 10.07 kg vs. PL: 3.42 ± 2.06 kg; *p* < 0.0001), leg press (GMCT: 29.32 ± 16.17 kg vs. PL: 5.21 ± 1.72 kg; p < 0.0001), number of leg extension repetitions (GMCT: 6.58 ± 2.57 vs. PL: 2.05 ± 1.22; p < 0.0001) in GMCT group were significantly improved, compared with placebo. Intergroup difference analyses show that the changes from baseline left arm (GMCT: 1.09 ± 0.36 cm vs. PL: 0.68 ± 0.42 cm; *p* = 0.0023), right arm (GMCT: 1.50 ± 0.44 cm vs. PL: 1.11 ± 0.43 cm; *p* = 0.0088) circumference and lean mass (GMCT: 2.29 ± 2.09 kg vs. PL: 0.52 ± 2.58 kg; *p* = 0.0404) in GMCT group were also significantly improved, compared with placebo. In comparison to placebo, GMCT supplementation did not improve free testosterone, IGF-1, insulin or lactate levels. Parameters of clinical biochemistry, hematology, urine and vital signs of the participants were within the normal range.

**Conclusion:**

GMCT supplementation is effective in increasing muscle strength, muscle size and, total lean mass, as well as endurance performance.

Trial Registration.

Clinical Trial Registry of India (CTRI/2015/01/005374), Registered on Jan 07, 2015; CTRI Website URL - http://ctri.nic.in

## Background

In nature, herbs are the major source of phytochemicals which have been used traditionally for various health benefits. Botanical products including herbal extracts or herbal compositions or concentrates are considered as dietary supplements [1]. Growing evidence from nutritional research has established that dietary supplements improve physical performance, accelerate post-exercise recovery, increase muscle mass and reduce body fat [2–4]. Biologically active phytochemicals modify the metabolic processes in the body to increase muscle power, enhance mental strength, and provide a physiological advantage [2]. The demand for safe and effective herbal dietary supplements among the professional and recreational athletes is growing extensively [4].

Earlier studies provided evidence in support of using nitric oxide boosters such as nitric oxide precursors, e.g., L-arginine, L-citrulline or dietary nitrates as nitric oxide substrates to increase physical performance and tolerance to exercise [5–10]. In a recent review article, Dominguez et al. summarized that chronic supplementation of beetroot juice as a source of nitrate improved the performances in resistance exercises [11]. Nevertheless, controversy exists whether exogenous sources of nitric oxide benefits physical performance in trained individuals [12]. A large pool of observations indicates that physical exercise enhances eNOS dependent NO synthesis and in cooperation with AMPK, nitric oxide up-regulates PGC1α and mitochondrial function to improve skeletal muscle function [13, 14]. An improved mitochondrial function is crucial to increase the aerobic respiratory capacity of the muscle cells. Increase in mitochondrial capacity is an essential factor to adapt and compensate the metabolic stress generated through endurance exercise and directly influence improving physical performance [15]. Given the importance of improved mitochondrial function for skeletal muscle performance, an attractive approach lies in augmenting endogenous nitric oxide pathway to enhance physical performance in conjunction with a training protocol.

We have developed a proprietary composition (GMCT) containing aqueous ethanol extracts of *Garcinia mangostana* fruit rind and *Cinnamomum tamala* leaf, which increases nitric oxide synthesis in human primary vascular endothelial cells via endothelial nitric oxide synthase (eNOS) activation (data not shown, to be communicated separately). *Garcinia mangostana* popularly known as mangosteen grows in southeast Asian countries such as India, Myanmar, Malaysia, Sri Lanka, and Thailand. The soft and juicy white fruit pulp is edible, slightly acidic and sweet with delightful flavor [16]. The mangosteen pericarp has been used for many years as a traditional medicine in treating sicknesses such as trauma, skin infection, abdominal pain, dysentery and wounds [17]. Mangosteen is rich in xanthones and α-mangostin is among the major xanthones present in the pericarp or rind [17]. α-mangostin is reported to provide several benefits including anti-inflammatory, analgesic, anti-oxidant, and anti-lipogenic activities [18]. *Cinnamomum tamala* or Indian bay leaf is native of India. The aromatic leaves have been used for culinary and medicinal purposes in Asian countries since ancient times. In Ayurveda, *C. tamala* leaves and bark have been used to treat rheumatism, cardiac disorders, colic, diarrhea, nausea and vomiting [19]. Moreover, *C. tamala* leaves are extensively used as a spice in the food industry [20], and as a natural food preservative [21]. The major phytochemicals present in *C. tamala* leaves are α-pinene, camphene, myrcene, limonene, p-cymene and other phenolic compounds [22, 23]. Extracts of *C. tamala* leaves or its essential oil has demonstrated potential anti-inflammatory, anti-oxidant, anti-microbial, anti-diabetic and hepatoprotective activities in vitro and in vivo models [22, 23].

In a separate in vitro study, the proprietary herbal blend GMCT activated eNOS in human vascular endothelial cells (results to be published elsewhere). Further, in vitro experiments showed that GMCT increased mitochondrial biogenesis and activated mTOR pathway in skeletal muscle cells (data not shown). Based on this background information, we hypothesized that GMCT supplementation in combination with a training program would improve physical performance and increase muscle strength of resistance trained individuals. Firstly, a proof of concept experiment was developed in a preclinical model of mice. Subsequently, a double-blind, placebo-controlled human trial was conducted on resistance-trained young, healthy males to validate the hypothesis.

The primary aim of this placebo-controlled trial was to determine the effect of GMCT supplementation in combination with a resistance training program on muscle strength and endurance. Also, we measured the clinical chemistry markers to evaluate the tolerability of GMCT supplementation as one of the secondary objectives of the study.

## Methods

### Study material

GMCT (LI80020F4 or CinDura®, Laila Nutraceuticals, Vijayawada, India) is a proprietary composition; it includes seven parts of an herbal blend containing aqueous ethanol extracts of *Garcinia mangostana* (GM) fruit rind and *Cinnamomum tamala* (CT) leaf at 1:2 ratio and three parts of the excipients. GMCT contains 28.1% (*w*/w) microcrystalline cellulose and 1.9% (w/w) Syloid as excipients. The final product was standardized to contain at least 3.5% α-mangostin and 0.1% rutin.

### Plant raw materials and extraction procedures

The plant raw materials viz. *G. mangostana* fruit rind and *C. tamala* leaves were procured from Indonesia and Nainital, Uttarakhand, India, respectively. A taxonomist identified the raw materials and the Taxonomy Division at Laila Nutraceuticals R&D Centre, Vijayawada, India preserved the voucher specimens of the plant materials. The voucher specimen numbers of *G. mangostana* and *C. tamala* are LNH6154 and LNH6300, respectively.

Typically, one kilogram of dried *G. mangostana* fruit rind pulverized to a coarse powder, extracted with 4 L aqueous ethanol (85% *v*/v) at reflux for one h. This extraction process was repeated two times under similar conditions. These extracts were combined, filtered, and concentrated to a thick mass containing more than 50% total solids, under vacuum at 60 °C. The final yield was 90 g of the powdered extract of *G. mangostana* rind.

For *C. tamala*, one kilogram of shade-dried leaves pulverized to a coarse powder and extracted with 6 L of aqueous ethanol (90% *v*/v) at 70 °C for one h. This extraction process repeated two times, and the combined extracts were filtered and evaporated under vacuum at 60 °C. The final yield was 125 g of *C. tamala* leaf extract.

### HPLC analysis

Analysis of GMCT was carried out using an ACUITY Ultra High-performance liquid chromatography system equipped with a thermostat controlled column oven compartment, autosampler, photodiode array detector and Empower 2 software (Waters Corporation, Milford, MA). The sample preparation involves extraction of the sample using aqueous-methanol, followed by filtration through 0.22 μm PVDF filter. The sample solution was analyzed using Waters X Bridge C18 column 3.5 μm (100 × 4.6 mm).

**Fig. 1:**
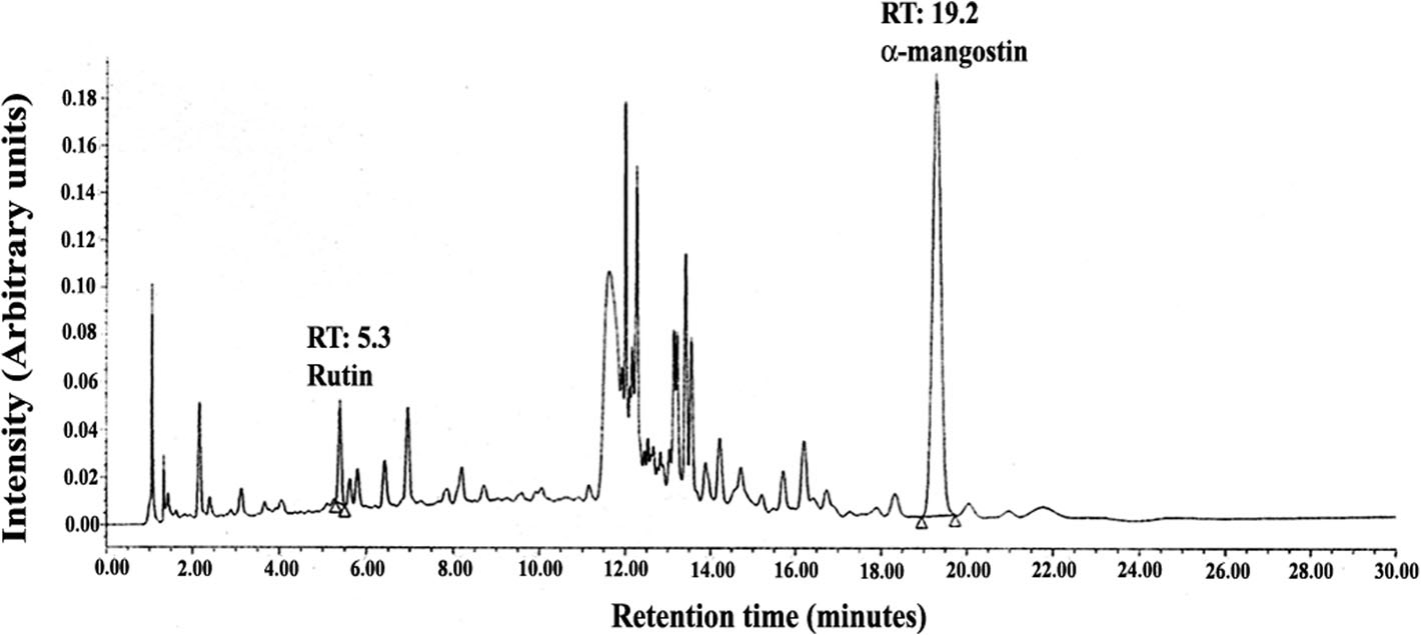
A typical HPLC chromatogram of GMCT showing peaks of Rutin and α-mangostin. The elution was detected at 255 nm. The elution profile is plotted in arbitrary units (AU) versus elution time (min). RT indicates retention time

A gradient elution system consists of solvent A (0.1% *v*/v Orthophosphoric acid in water) and solvent B (Acetonitrile) as mobile phases with a flow rate of 1.0 ml/min. The run started at sample injection with a mixture of 90% A and 10% B as initial eluent. Then a linear gradient was used to reach 70% A and 30% B in 10 min; then up to 35% A, 65% B in 1 min; finally maintained isocratic run at 35% A, 65% B for 11 min. The column oven compartment was maintained at 40 °C. Figure 1 depicts a typical HPLC chromatogram of GMCT. The chromatographic profile at 255 nm shows two peaks at 5.3 and 19.2 min, which represent rutin and α-mangostin, respectively. Identification of these peaks was carried out using individual reference standards (Sigma-Aldrich, St. Louis, MO).

### Preclinical proof of concept study

#### Animals

Male Swiss albino mice of 8–10 weeks old (20–30 g body weight) were purchased from Palamur Biosciences Pvt. Ltd. (Hyderabad, India). The animals were housed under standard environmental conditions at 22 ± 2 °C, 50–70% relative humidity and in 12 h light/dark cycle during the study. Mice were given pellet feed (Labmeal™-M, Indian Immunologicals Pvt. Ltd., Rajkot, India) and drank water ad libitum. The Institutional Animal Ethics Committee (IAEC) approved the study protocol (approval no. LI/IAEC/LI170606). Animal care and study procedure complied with the guidelines specified by the Committee for the Purpose of Control and Supervision of Experiments on Animals (CPCSEA) and OECD.

#### Treatments

The mice were acclimatized to the laboratory conditions for one week before the experiment. Then, the animals were assigned randomly into four groups (*n* = 6). The animals in different groups were treated with either 0.5% carboxymethylcellulose sodium (CMC-Na) (CMC; vehicle control) or 150 mg/kg GMCT (GMCT-150) or 300 mg/kg GMCT (GMCT-300). In parallel, 50 mg/kg Oxymetholone treated group (OXY) was used as an active control group. Oxymetholone (Anadrol) is an anabolic androgenic steroid, improves muscle mass and muscle strength; and is being used to enhance sports performance [24]. The treatment doses of the test product and Oxymetholone were determined based on a pilot study (data not shown). The treatments were administered through oral gavage once a day for 21 days.

#### Forced swim test (FST)

The forced swim test (FST) was conducted in a cylindrical acrylic tank (20 cm diameter, 45 cm height), filled with warm water at 36 ± 2 °C, following the method described earlier [25]. A load of 5% of the body weight of each mouse was bound to their tail and, they were forced to swim for 10 min. Swimming parameters such as resting time, swimming time, total distance covered and average velocity were recorded and analyzed by the SMART video tracking system (PanLab S.L.U., Holliston, MA).

#### Grip strength measurement

The forelimb grip strength of mice was evaluated using a digital grip strength meter (Laboratory Enterprises, Nasik, India) following the method described earlier [26]. Each animal was trained for 2–3 days for familiarization with the experimental conditions. The mice were allowed to grasp the grasping bar equipped with a force transducer and were pulled back horizontally by their tail. The maximum force applied to release the grasp from the bar was recorded as grip strength. Three trials were conducted, and average grip strength was taken from each animal. The grip strength values acquired from each animal was normalized with their respective body weights.

#### Muscle morphometry

Gastrocnemius muscle tissue samples were fixed in 10% neutral buffered formalin for 24 h. The paraffin-embedded tissues were cut into 5 μm sections, and those were processed following a standard protocol [27]. The hematoxylin-eosin stained tissue sections were analyzed under a microscope at 40X (Axio Scope A1, Carl Zeiss GmbH, Jena, Germany). The bright field images were captured using a CCD camera (ProgRes C5, Genoptik, Jena, Germany). Pixel sizes of the muscle fibers were estimated using Axiovision 4.8 software (Carl Zeiss GmbH, Jena, Germany). A conversion scale of 0.1694 μm per pixel was used to determine the area of each fiber and represented in μm^2^.

### Clinical study

#### Study design

A randomized, double-blind, placebo-controlled, parallel group study was conducted to evaluate the efficacy of GMCT on increasing muscle strength and endurance of exercise in resistance-trained healthy male subjects. This study followed the ICH-GCP guideline. The Institutional Ethics Committee of Alluri Sitarama Raju Academy of Medical Sciences (ASRAM), Eluru, India approved the study protocol by (Ref. No. IEC/ASR/001/15). The trial was registered in the Clinical Trial Registry of India (CTRI no. CTRI/2015/01/005374).

Thirty-eight resistance-trained (> 6 months) male subjects completed the study. The subjects aged between 19 and 39 years (mean 26.32 ± 4.39 years), with body weight 67.79 ± 12.84 kg and BMI 22.92 ± 3.54 kg/m^2^. They had normal health status, based on clinical laboratory examination. They were practicing standard resistance exercises for the lower and upper portion of the body at a frequency of 2–3 days a week. The subjects were familiar with the exercises included in the training protocol of the study. The objective and procedure of the study were explained to the subjects. They written consent of willingness to join the study. The subjects were excluded if they met any of the following criteria: (i) taking any medication or steroids or ergogenic nutritional supplements (creatine, arginine, citrulline, HMB, proteins, etc.); (ii) history of asthma, cardiovascular diseases; (iii) diabetes, thyroid dysfunction, abnormal liver or kidney functions; (iv) history of any hematological disorders; (v) history of drug abuse or consuming more than 2 standard alcoholic drinks daily.

The enrolled subjects were randomized into two groups, viz. Placebo and GMCT. Each group contained twenty subjects. The participants were advised to maintain their regular dietary habits and to refrain from consuming any nutritional supplements or energy drinks containing creatine, arginine, citrulline, HMB, proteins or amino acids during the intervention. The participants were also instructed that they should maintain their regular diet plan during the study. The study coordinator routinely inquired whether they adhered to the instruction.

**Fig. 2:**
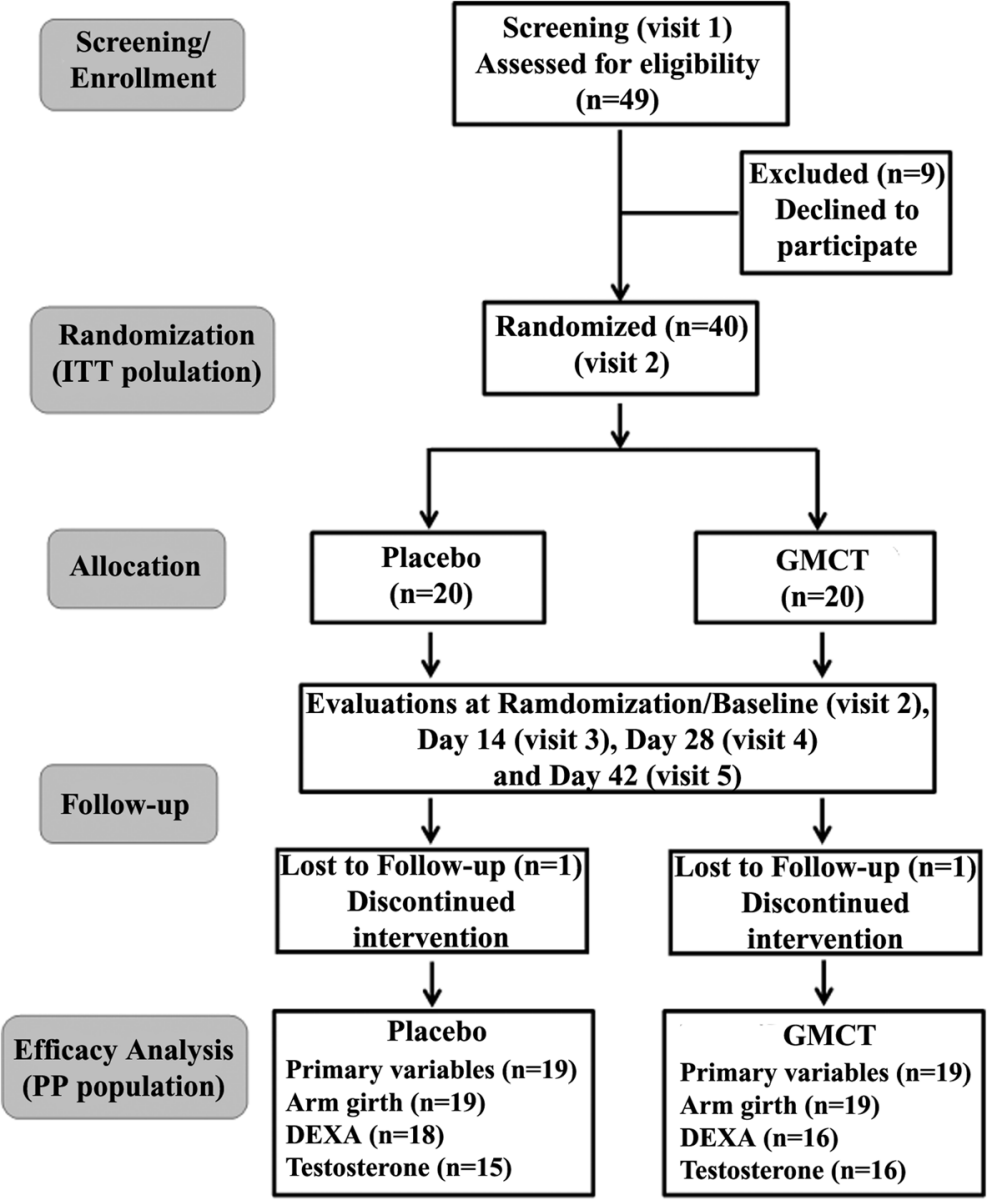
The Consort flow diagram presents enrollment, randomization, follow-up, and analysis of the double-blind placebo controlled parallel group trial

The subjects received two capsules of either placebo or 400 mg GMCT daily for 42 days. Each placebo capsule contained 400 mg excipients, and it was identical with the GMCT capsule in physical appearance. The study consisted of screening visit (visit 1), randomization visit or baseline (visit 2), first follow up visit at day 14 (visit 3), second follow up visit at day 28 (visit 4) and the final visit at day 42 (visit 5). The subjects received the capsules in coded and sealed bottles at baseline, day 14, and at day 28. They returned the capsules that were not consumed, to the study coordinator. Figure 2 summarizes subject enrollment, allocation, follow-ups, and analysis. Primary efficacy variables were the changes in baseline scores of muscular strength and endurance; these were estimated through the 1-RM strength and number of repetitions in leg extension exercise, respectively in GMCT supplemented subjects in comparison with those of placebo. Besides, this study also evaluated the effect of the herbal supplement on the mid-upper arm circumference, changes in body composition using Dual-energy X-ray absorptiometry (DEXA). Mid-upper arm circumference was measured using a measuring tape placed tightly around the relaxed bicep at the midpoint between the shoulder and the tip of the elbow. Subjects were instructed not to perform the exercise for 24 h and fast for 12-h before each evaluation visit including baseline.

#### Training protocol

The resistance-trained subjects had a practice schedule of 5 days a week during the study. The subjects performed seventeen exercises with 2 min rest between two sets. Initially, the subjects warmed up at 50% of their 1-RM in two sets of 8–10 repetitions. Then they performed 2–3 sets of 10 repetitions at 70% of the baseline 1-RM. Following successful completion of 10 repetitions at 70% of 1-RM, the resistance was successively increased up to 90% of the baseline 1-RM. They performed 2–3 sets of 10 repetitions at each load level of each exercise. Between two exercises the participants took 10 min rest. The practice schedules 1, 2, 3 and 4 contained the exercises for chest/shoulder, back, legs, and the upper arms, respectively. The participants performed each schedule in one day, and they continued on the following days in a sequence. The maintained the series through the study period. The subjects performed resistance training exercise for chest/shoulder (incline dumbbell press, flat barbell press, dumbbell shoulder press, lateral raise), back (bent rows, cable pulldowns, one arm dumbbell, seated rear deltoid raises), legs (calf raises, leg extension, lying leg curls, leg press) and arms (EZ-bar curl, alternating dumbbell curls, overhead dumbbell extensions, hammer curls, kick back/cable push down). The local gyms conducted the training programs under the supervisions of trained instructors and the study personnel. The study personnel recorded the information such as weight lifted, number of repetitions after each set in the training log for each subject.

#### One-repetition maximum (1-RM) test

Change in muscular strength was the primary efficacy variable of the study. Upper and lower body muscle strength were assessed performing one-repetition maximum (1-RM) at a supine bench press and in seated leg press, respectively. Both 1-RM exercises were performed following the recommendations of the National Strength and Conditioning Association [28]. Briefly, in bench press 1-RM, the subjects warmed-up by lifting approximately 50% of anticipated maximum weight for 8–10 times. After a 2-min rest period, a set of 2–3 repetitions was performed in 60–80% of the participant’s perceived 1-RM. Subsequently, 3–5 maximal trials (one repetition sets) were conducted to determine the 1-RM. Following a rest period of 10 min, the subjects warmed-up on 45^o^ leg press by lifting 50% of their maximum anticipated weight for 8–10 times. Similarly, the subjects lifted the weights successively in 10% increments until they reached the 1-RM on the leg press.

Blood pressure (BP) and Pulse rate were monitored by the oscillometric method (Omron Automatic blood pressure monitor; Model HEM 7112, Vietnam) before each test at each visit during the trial.

#### Leg extension

The subjects performed the leg extension exercise on a leg extension machine (Power Center Combo Bench, Body-Solid Inc., Forest Park, IL) to evaluate their strength endurance. The participants lifted a fixed weight and held the pressure in the same position for one second. They were advised to push the pad against the weight by their feet from a starting angle of 90^o^ between lower and upper leg to a position of the completely flexed lower leg, and slowly to return to the starting position. The weight was fixed for a participant based on 70% of his maximum lift capacity. Each lift from start to finish took between four and five seconds. The subjects repeated the lift until they reached exhaustion.

#### Serum markers

Blood samples were drawn from the participants at baseline and at the end of the study for measuring free testosterone, lactate, insulin, and IGF-1 in serum. The blood draw was conducted before 9 AM and before starting any exercise to avoid the diurnal effect and exercise-related influence on testosterone level, respectively. The free testosterone was measured using a commercial enzyme immunoassay (EIA) kit (DRG International Inc., Springfield Township, NJ). The assay was performed following the vendor’s instructions. Each serum sample was run in duplicate wells. The assay utilized competitive immuno-enzymatic reactions, and the color development was recorded at 450 nm in a microplate reader (Bio-Rad Laboratories, Hercules, CA). The sensitivity of the kit was 0.06 pg/ml.

Serum insulin and IGF-1 were estimated using commercial ELISA kits, procured from BioVision Inc. (Milpitas, CA). The assays were performed following the vendors’ instructions. The minimum detectable concentrations of insulin and IGF-1 were less than 4 μIU/ml and 0.1 ng/ml, respectively. Serum lactate was measured using a commercial kit following a protocol recommended by the vendor (BioVision Inc., Milpitas, CA). The kit utilizes a principle of converting lactate to its oxidized form, which interacts with a probe to produce a color (λmax at 450 nm). The minimum detectable concentration of lactate was 0.02 mM.

Subjects’ body compositions were analyzed using DEXA (QDR Explorer™, Hologic, Bedford, MA) at baseline and at the end of the study. Anthropometric measurements such as body weight, height and arm circumference (flexed mid upper-arm) were measured using standard calibrated tools. The safety parameters including clinical biochemistry, hematology, urine analysis, and vital signs were measured at baseline and the end of the study. In clinical biochemistry, fasting glucose, serum creatinine, uric acid, blood urea nitrogen, serum bilirubin, ALT, AST, serum alkaline phosphatase, sodium, potassium, serum albumin; in hematology, hemoglobin, platelet count, total leukocyte count, RBC, ESR, differential count; and in routine urine analysis, color, specific gravity, pH, glucose, protein, RBC were performed. Also, the critical vital signs such as blood pressure (systolic/diastolic), pulse rate, respiratory rate, and oral temperature were evaluated.

### Statistical analysis

The unpaired t-test was used to analyze the preclinical efficacy data, comparing treatment vs. vehicle control group. The clinical safety and efficacy data were analyzed on intention-to-treat and per-protocol bases, respectively. The primary comparison was the evaluation of mean change from baseline to end of the study using Student’s t-test. Mean change in treatment group vs. placebo at all evaluations were analyzed using ANCOVA on safety and efficacy parameters. Wherever necessary an additional t-test was used to compare two efficacy datasets. Results with *p* < 0.05 were considered statistically significant.

It was estimated that at least 20 subjects in each arm would provide greater than 90% power to detect a treatment difference for the primary efficacy variable at a two-sided significance level of 0.025%. The sample size was calculated assuming a mean difference of 11 with a common standard deviation of 10.2.

## Results

### Swimming performances of *Swiss albino* mice

**Fig. 3:**
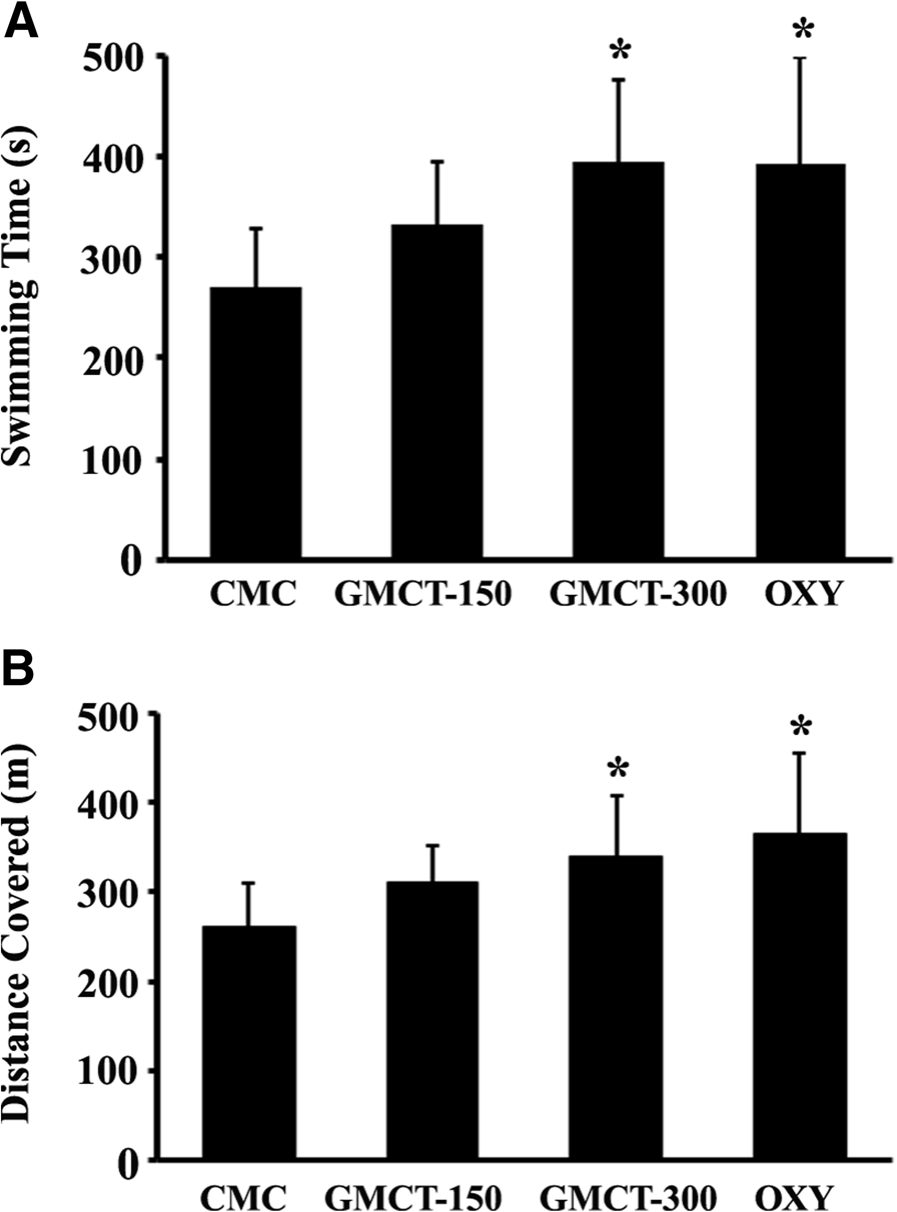
GMCT improves swimming parameters of the weight-loaded mice in a Forced Swim test. Swimming time (**a**) and the total distance covered (**b**) are graphically presented. The groups presenting CMC, GMCT-150, GMCT-300, and OXY are described in Methods. Each bar represents mean ± SD (*n* = 6). *p < 0.05 compared with the vehicle control group (CMC), using unpaired t-test

Twenty-one days GMCT supplementation increased the swimming time and reduced immobilization or resting time within the stipulated 600 s performance period, in comparison with the mice in the control group (Fig. 3a). On average, the mice assigned to the 150 and 300 mg GMCT groups swam 22.67% (*p* = 0.1225; 333.17 ± 62.70 s vs. 271.6 ± 56.86 s in control) and 45.54% (*p* = 0.0166; 395.3 ± 81.70 s vs. 271.6 ± 56.86 s in control) longer than the CMC-Na supplemented control animals (Fig. 3a). In addition, low and high dose of GMCT supplemented animals covered 19.67% (*p* = 0.0994; 312.18 ± 40.29 m vs. 260.84 ± 49.15 m) and 30.81% (*p* = 0.0461; 341.22 ± 65.88 m vs. 260.84 ± 49.15 m) longer distance than the control animals, respectively (Fig. 3b). Together, these observations suggest that the animals in GMCT-300 group gained significant strength endurance than the control animals.

As anticipated, the Oxymetholone (OXY) supplemented group exhibited 44.72% increase in swimming time (*p* = 0.0434; 393.07 ± 106.90 s vs. 271.6 ± 56.86 s in control) and covered 40.34% (*p* = 0.0389; 366.08 ± 89.52 m vs. 260.84 ± 49.15 m in control) more distance, in comparison with the control animals (Fig. 3a). However, these improvements in the OXY group were not statistically different when compared with the GMCT-150 (*p* = 0.2701, swimming time; *p* = 0.2209, distance covered) and the GMCT-300 (*p* = 0.9684, swimming time; *p* = 0.5968, distance covered).

### Grip strength and muscle fiber size in *Swiss albino* mice

**Fig. 4:**
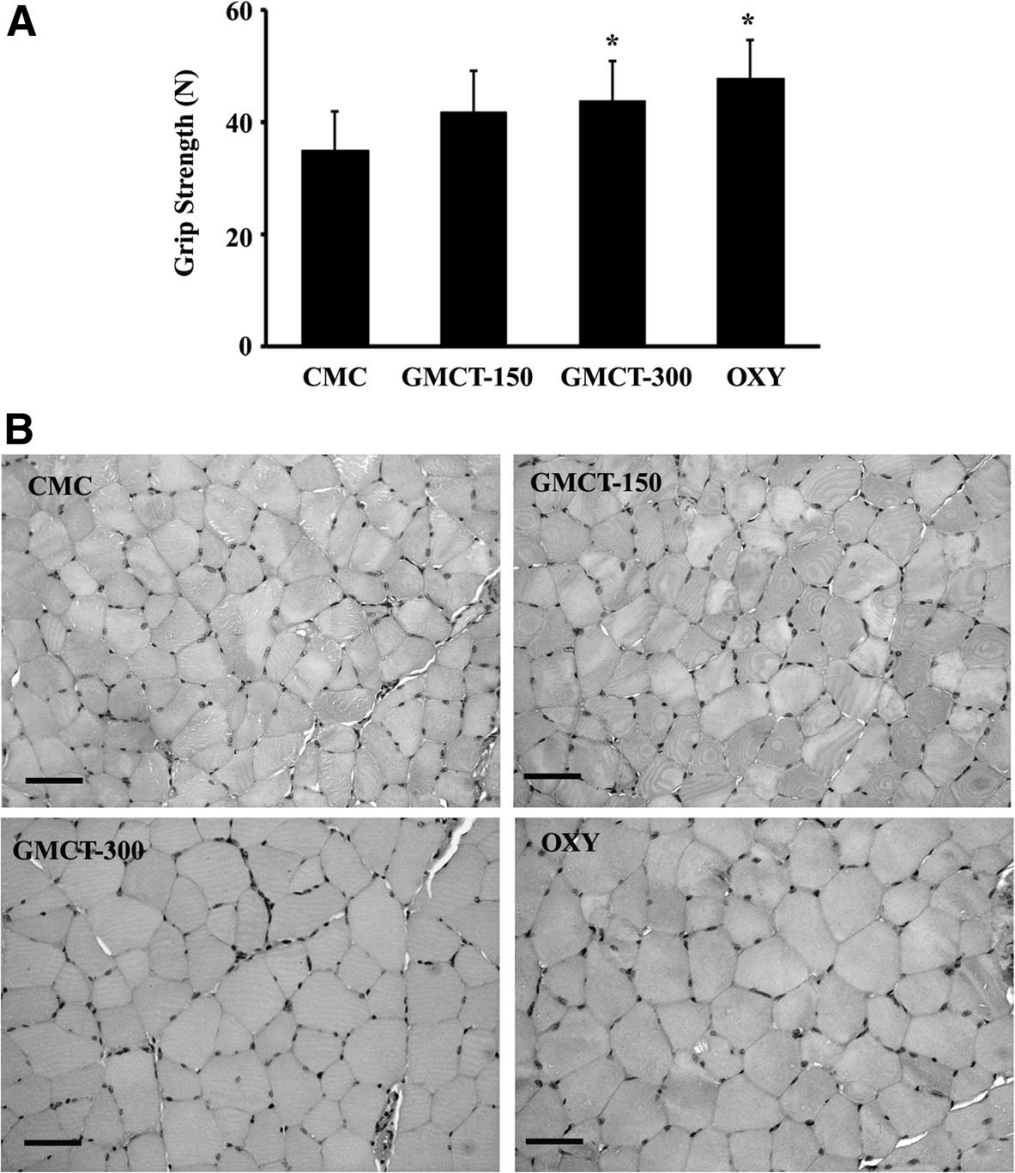
GMCT positively modulates muscle parameters in mice. The groups presenting CMC, GMCT-150, GMCT-300 and OXY are described in Methods. **a** Bar diagram represents the change in grip strength in experimental animals (n = 6). Each bar represents mean ± SD of strength in Newton per gram of body weight as mentioned in Results. *p < 0.05 compared with the vehicle control group (CMC), using unpaired t-test. **b** Representative photomicrographs show Hematoxyline-Eosin stained cross-sections of the gastrocnemius muscles of the different group of animals, as indicated. Bar indicates 50 μm

Grip strength measurement demonstrates that herbal supplementation positively influences muscle strength in the experimental animals (Fig. 4a). One of the factors which influence the grip strength is the body weight. Thus, the recorded grip strength scores were normalized with the respective animal’s body weight. Here, we present the normalized grip strength in Newton per gram of body weight. Supplementation of 150 and 300 mg of GMCT increases muscle strength by 19.66% (*p* = 0.0956; 41.88 ± 7.20 vs. 35.0 ± 6.92 in control) and 25.49% (*p* = 0.0490; 43.92 ± 6.97 vs. 35.0 ± 6.92 in control), respectively (Fig. 4a). In parallel, Oxymetholone also showed significant improvement (36.37%, *p* = 0.009) in muscle strength in the experimental animals. However, this effect in the OXY group was not statistically different when compared with the GMCT-150 (*p* = 0.1566) and with the GMCT-300 (*p* = 0.2729).

Further, in morphometric analyses, we observed that 150 and 300 mg of GMCT supplementation for 21 days resulted in 15.20% (*p* = 0.4682; 1483.51 ± 222.34 μm^2^ vs. 1287.65 ± 281.35 μm^2^ in control) and 16.09% (*p* = 0.2890; 1494.88 ± 328.53 μm^2^ vs. 1287.65 ± 281.35 μm^2^ in control) increase in gastrocnemius muscle fiber sizes, respectively. In parallel, Oxymetholone treatment also demonstrated a 20.63% (*p* = 0.1203; 1553.29 ± 202.78 μm^2^ vs. 1287.65 ± 281.35 μm^2^ in control) increase in muscle fiber size (data not shown). Figure 4b depicts representative photomicrographs of the hematoxylin-eosin stained cross-sections of the gastrocnemius muscle tissues.

### Strength and endurance in resistance trained males

Descriptive analysis of the baseline demographic variables is presented in Table 1. Each arm of the trial consisted of 20 resistance-trained young men. The average age, body weight and BMI of the entire study population were 26.28 ± 4.30 yrs., 67.48 ± 12.59 kg and 22.82 ± 3.48 kg/m^2^, respectively. Following randomization, the comparative analyses of the baseline characteristics suggest that the placebo and GMCT group are not statistically different (Table 1).

**Table 1:** Baseline demographic characteristics of the participants in placebo and GMCT supplemented groups

Characteristics/Parameters	Placebo (*n* = 20) Mean ± SD	GMCT (n = 20) Mean ± SD	*p* value*
Age (yrs.)	26.95 ± 3.35	25.60 ± 5.25	0.3381
Body weight (kg)	68.64 ± 11.27	66.33 ± 13.91	0.5672
BMI (kg/m^2^)	22.89 ± 3.13	22.75 ± 3.82	0.9034
1-RM Bench Press (kg)	48.55 ± 8.78	49.20 ± 8.79	0.8163
1-RM Leg Press (kg)	68.30 ± 30.61	72.45 ± 21.36	0.6219
No. of repetitions -leg extension	4.30 ± 1.30	4.35 ± 3.34	0.9506
Left arm circumference (cm)	32.92 ± 4.86	33.38 ± 2.44	0.7158
Right arm circumference (cm)	34.00 ± 5.03	33.80 ± 2.19	0.8746
Lean Body Mass (kg)	51.58 ± 7.86	50.05 ± 8.46	0.5902
Total Body Fat (kg)	17.24 ± 7.29	17.75 ± 5.98	0.8242
Total Body Fat (%)	23.58 ± 7.84	24.87 ± 4.11	0.5485

*Comparison between Placebo and GMCT groups using unpaired t-test, assuming equal variances (p < 0.05)

The number of training sessions completed by the participants was used to estimate the subjects’ adherence to training. Out of 30 training sessions, the participants in the placebo and GMCT group completed 28.9 ± 1.32 and 29.4 ± 1.08 sessions (*p* = 0.2332), respectively. Overall, the adherence to the training sessions by the participants was 97.1%. The percent of capsules consumed by the participants was used to estimate the adherence to treatment. The placebo and GMCT supplemented subjects consumed 95.7 ± 5.64% and 94.1 ± 7.35% of capsules (*p* = 0.4589), respectively.

Table 2 shows the changes in 1-RM bench and leg press performance over the course of the 42-day trial period. At the end of the trial, GMCT supplemented group experienced 47.69% (23.47 ± 10.07 kg) and 39.83% (29.32 ± 16.17 kg) improvements in the 1-RM bench press and 1-RM leg press, respectively from baseline. In contrast, the placebo group experienced 7.09% (3.42 ± 2.06 kg) and 7.68% (5.21 ± 1.72 kg) increase from baseline 1-RM bench press and leg press, respectively (Table 2). At post-intervention, the inter-group analyses between the mean changes from baseline scores reveal that the herbal supplementation provides significant improvements in the 1-RM bench press ((*p* < 0.0001; 23.47 ± 10.07 vs. 3.42 ± 2.06 kg) and leg press (p < 0.0001; 29.32 ± 16.17 vs. 5.21 ± 1.72 kg) (Table 2). Gradual improvements of the baseline 1-RM in the GMCT group are evident during the intervention period (Table 3). Intragroup analyses show that GMCT supplementation for early 14 days results in significant improvements in the 1-RM bench press (55.47 ± 8.75 kg vs. 49.21 ± 9.03 kg, *p* < 0.0001) and the leg press (82.84 ± 20.79 kg vs. 73.58 ± 21.33 kg, *p* < 0.0001) from baseline. The improvements in the 1-RM bench press (49.00 ± 8.60 kg vs. 48.21 ± 8.89 kg, *p* = 0.1034) and the leg press (69.00 ± 30.73 kg. vs. 67.79 ± 31.36) in the placebo group are not significant (Table 3). Intergroup analyses also show that the GMCT supplementation resulted in significant improvements in the 1-RM bench press (6.26 ± 2.60 kg vs. 0.79 ± 1.18 kg in placebo, *p* < 0.0001) and the leg press (9.26 ± 5.36 kg vs. 1.21 ± 1.23 kg, *p* < 0.0001 in placebo) as early as 14 days from the start of the intervention (Table 2).

**Table 2:** Per protocol analysis of mean changes from baseline scores of exercise performances in placebo vs. GMCT supplemented subjects

Parameters	Groups	Mean Change from baseline at					
		Day 14		Day 28		Day 42	
		Mean ± SD	*p* value*	Mean ± SD	*p* value*	Mean ± SD	*p* value*
1-RM Bench Press (kg)	Placebo (*n* = 19)	0.79 ± 1.18	< 0.0001	2.21 ± 1.62	< 0.0001	3.42 ± 2.06	< 0.0001
	GMCT (*n* = 19)	6.26 ± 2.60		18.89 ± 8.44		23.47 ± 10.07	
1-RM Leg press (kg)	Placebo (*n* = 19)	1.21 ± 1.23	< 0.0001	3.21 ± 1.62	< 0.0001	5.21 ± 1.72	< 0.0001
	GMCT (*n* = 19)	9.26 ± 5.36		21.53 ± 15.06		29.32 ± 16.17	
No. of repetitions -Leg extension	Placebo (*n* = 19)	1.16 ± 0.50	< 0.0001	1.79 ± 0.71	< 0.0001	2.05 ± 1.22	< 0.0001
	GMCT (*n* = 19)	3.00 ± 1.41		5.11 ± 2.40		6.58 ± 2.57	

*Comparison analyses between changes from baseline in placebo vs. GMCT on respective days of evaluation using ANCOVA (p < 0.05)

**Table 3:** Gradual improvements of the strength and endurance parameters in placebo and GMCT supplemented subjects during the study

Parameters	Evaluations	Placebo (*n* = 19)		GMCT (n = 19)	
		Mean ± SD	*p* value*	Mean ± SD	*p* value*
1-RM Bench press (kg)	Baseline	48.21 ± 8.89	–	49.21 ± 9.03	–
	Day 14	49.00 ± 8.60	0.1034	55.47 ± 8.75	< 0.0001
	Day 28	50.42 ± 8.38	0.1189	68.11 ± 8.82	< 0.0001
	Day 42	51.63 ± 8.62	0.0471	72.68 ± 10.28	< 0.0001
1-RM Leg press (kg)	Baseline	67.79 ± 31.36	–	73.58 ± 21.33	–
	Day 14	69.00 ± 30.73	0.2152	82.84 ± 20.79	< 0.0001
	Day 28	71.00 ± 30.40	0.2134	95.11 ± 26.42	< 0.0001
	Day 42	73.00 ± 30.14	0.0660	102.89 ± 25.20	< 0.0001
No. of maximum repetitions-Leg press	Baseline	4.26 ± 1.33	–	4.16 ± 3.32	–
	Day 14	5.42 ± 1.12	< 0.0001	7.16 ± 2.99	< 0.0001
	Day 28	6.05 ± 0.97	< 0.0001	9.26 ± 2.79	< 0.0001
	Day 42	6.32 ± 1.11	< 0.0001	10.74 ± 2.56	< 0.0001

*Intragroup comparison between baseline and respective days of evaluation in placebo and GMCT group using t-test (p < 0.05)

The number of repetitions performed during the leg extension exercise was a measure of endurance. In the leg extension exercise, at post-intervention, GMCT group performed 6.58 ± 2.57 more repetitions; whereas, placebo performed 2.05 ± 1.22 more repetitions from baseline (Table 2). Comparative analysis of baseline and post-intervention data indicates GMCT supplementation significantly increased (p < 0.0001) endurance performance. After the initial 14 days of intervention, intragroup analyses show that the GMCT group performed a substantially higher number of maximum repetitions from baseline (7.16 ± 2.99 from 4.16 ± 3.32; p < 0.0001). In parallel, the subjects in placebo also performed a significantly higher number of maximum repetitions from baseline (5.42 ± 1.12 from 4.26 ± 1.33, p < 0.0001) (Table 3). Intergroup analysis reveals that consistent with the substantial increase in 1RM strength; the GMCT group also showed an increase in the maximum number of repetitions (p < 0.0001; 3.00 ± 1.41 vs. 1.16 ± 0.50 in placebo) after 14 days of intervention (Table 2).

### Mid-upper arm circumference

**Fig. 5:**
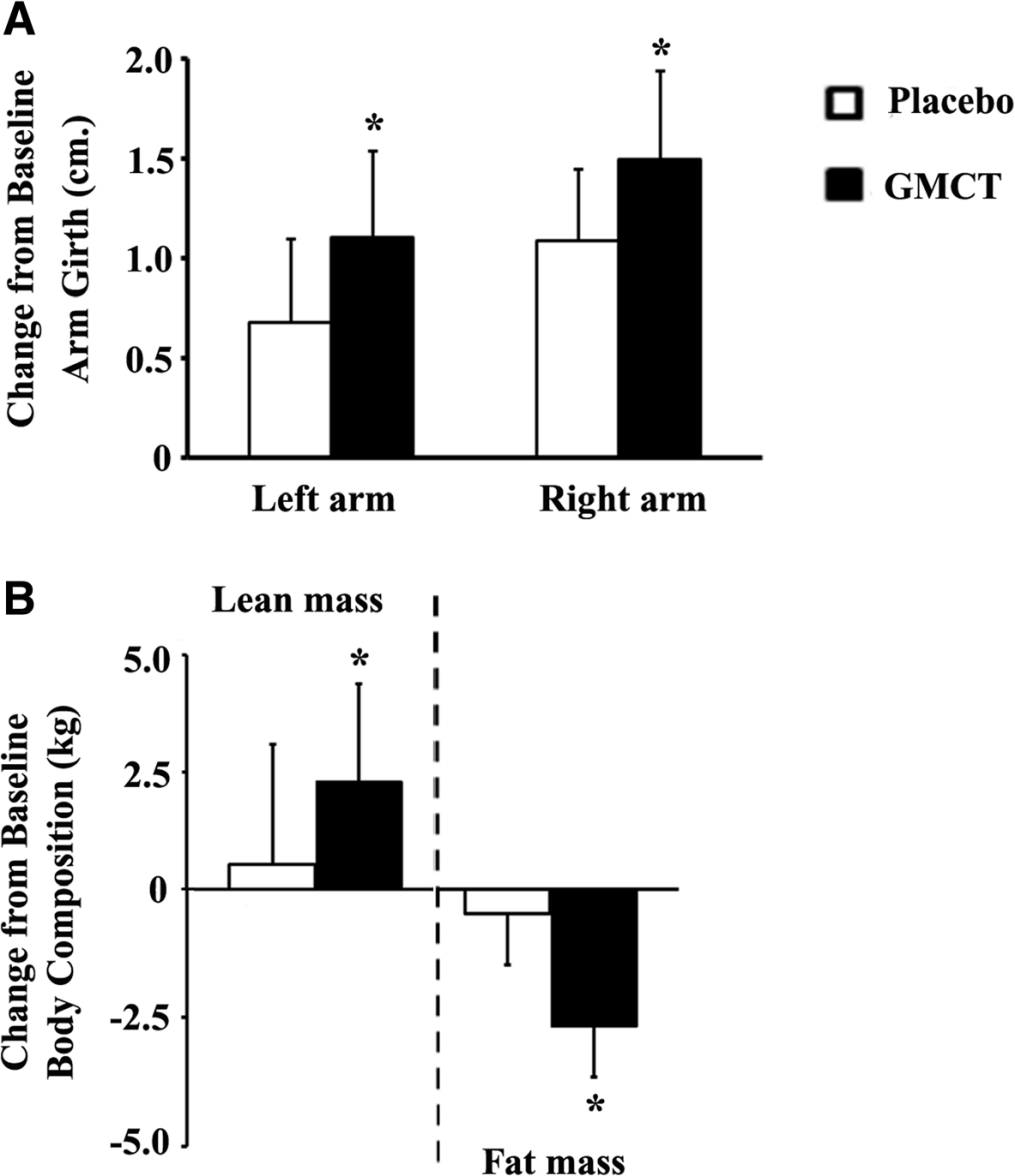
GMCT supplementation improves anthropometric measurements in resistance-trained participants. **a** Changes from baseline mid-upper arm circumference (placebo, *n* = 19; GMCT, *n* = 19) and (**b**) changes from baseline body composition (placebo, *n* = 18; GMCT, *n* = 16) at post-intervention are graphically presented. Each bar represents mean ± SD. *p < 0.05, comparison between changes in placebo and GMCT using ANCOVA

Figure 5a presents the changes in mid-upper arm circumference (MUAC) before and after the intervention. Typically, MUAC measurement refers to monitoring the growth of the biceps brachii and triceps brachii muscles. At the end of the study, the participants in the GMCT group recorded bigger increases in arm girth from baseline, in comparison with the change in placebo. From baseline, the left and right arm circumferences of GMCT supplemented subjects were increased by 1.09 ± 0.36 cm (*p* = 0.0023; vs. 0.68 ± 0.42 cm. in placebo) and 1.50 ± 0.44 cm (*p* = 0.0088; vs. 1.11 ± 0.43 cm. in placebo), respectively (Fig. 5a).

### Body composition

Figure 5b depicts the mean changes in lean body mass and fat mass of the participants in both groups at the end of the intervention (from baseline). Among the completers, one participant in the placebo and three participants in the GMCT group declined to take part in the DEXA scan at the final day of the intervention. At post-intervention, the baseline lean body mass in the GMCT group (*n* = 16) was increased by 2.29 ± 2.09 kg (50.05 ± 8.46 kg and 52.34 ± 8.25 kg at baseline and day 42, respectively); in contrast, the lean mass of the subjects in the placebo group (*n* = 18) increased by 0.52 ± 2.58 kg from baseline (51.58 ± 7.86 kg and 52.11 ± 8.76 kg at baseline and day 42, respectively). Intergroup comparisons between the baseline changes showed significant improvement (*p* = 0.0404) of lean body mass in GMCT supplemented group, in contrast with the placebo.

In parallel, the GMCT group showed a reduction of 2.69 ± 2.72 kg fat mass (17.75 ± 5.98 kg and 15.06 ± 6.10 kg at baseline and day 42, respectively); in contrast, the fat mass of the subjects in the placebo group reduced by 0.48 ± 3.36 kg (17.24 ± 7.29 kg and 16.76 ± 5.81 kg at baseline and day 42, respectively). Intergroup comparison between the baseline changes showed a significant reduction (*p* = 0.0350) of fat mass in GMCT supplemented group, in comparison with the placebo (Fig. 5b).

### Serum testosterone

**Fig. 6:**
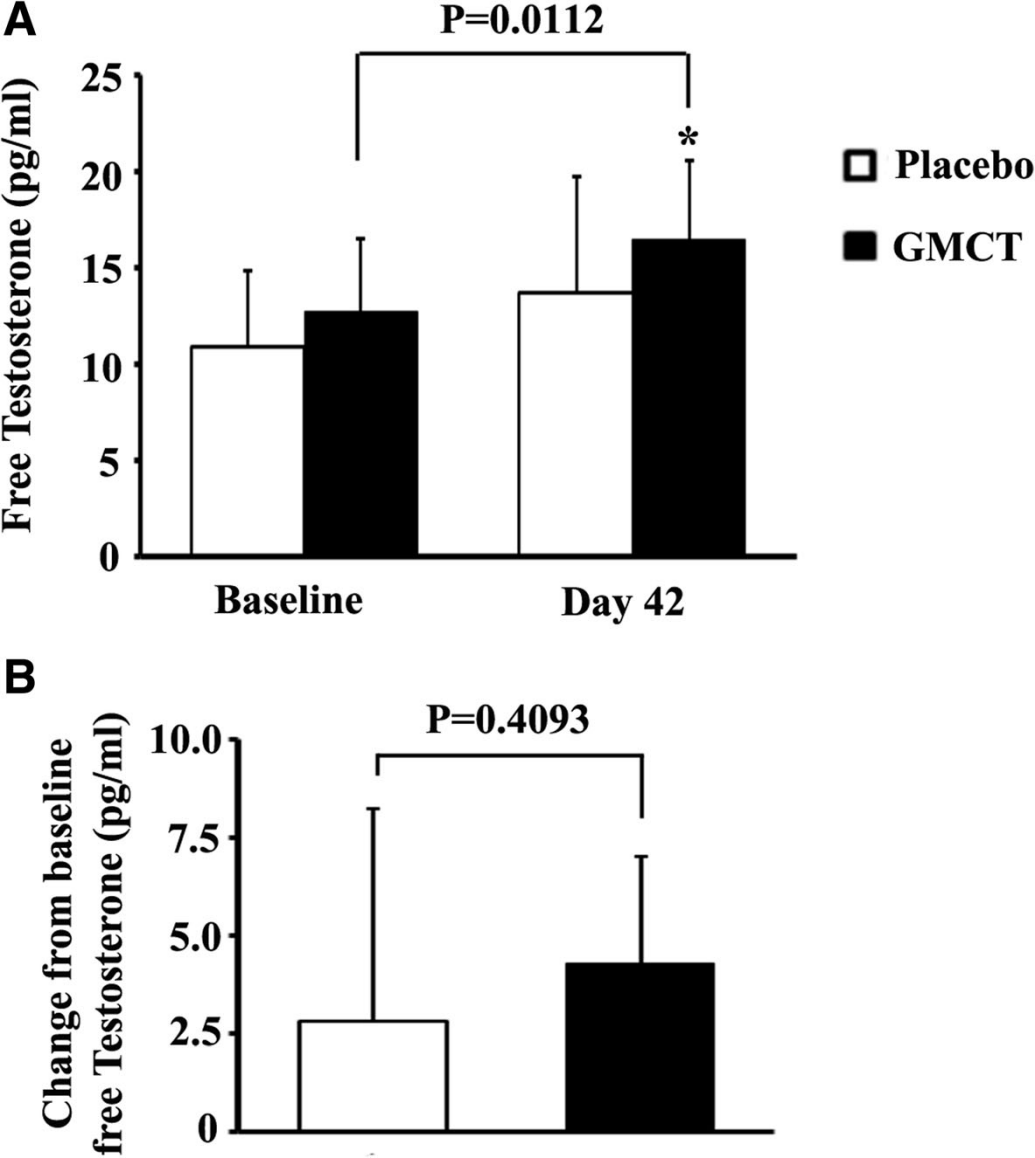
Effect of GMCT supplementation on free testosterone level in the circulation of resistance-trained participants. **a** Bars represent mean ± SD of free testosterone concentrations in placebo (*n* = 15) and GMCT (n = 16) groups at baseline and post-intervention. **b** Bars represent mean ± SD of changes from baseline free testosterone concentrations in placebo and GMCT groups at post-intervention. * indicates significance in an unpaired t-test

The hemolysed serum samples were excluded from performing the assay; fifteen samples from placebo and sixteen samples from GMCT group were analyzed for the serum markers. After 42 days intervention, free testosterone concentration in serum of the GMCT group was significantly improved compared to baseline (*p* = 0.0112; 16.52 ± 4.09 from 12.74 ± 3.82 pg/ml; 32.39% increase) (Fig. 6a). In parallel, the free testosterone level of the subjects in placebo group increased as well (13.71 ± 6.01 from 10.88 ± 3.99 pg/ml; 26.01% increase), but the difference did not reach statistical significance (*p* = 0.1394). At the end of the intervention, the intergroup comparison showed that the improvements in baseline free testosterone level in GMCT and placebo groups were 4.29 ± 2.73 pg/ml and 2.83 ± 5.39 pg/ml, respectively. The intergroup difference was not statistically significant (*p* = 0.4093) (Fig. 6b).

At post-intervention, the changes from baseline insulin, IGF-1 and lactate levels within the groups were minimal and were not statistically different. Also, the intergroup comparisons between the mean changes from baseline insulin, IGF-1 and lactate levels were highly insignificant (data not shown).

### Safety parameters

In addition to the efficacy, we also conducted a battery of routine clinical tests to evaluate the tolerability and safety of GMCT. The parameters tested in serum biochemistry, hematology, urine analysis, and vital signs are mentioned in the Methods. At the screening visit, all tested parameters were within typical normal range. None of the parameters in the study samples showed any abnormal changes at the end of the intervention (data not shown). In addition, vital signs and urine analysis parameters were also within the normal range throughout the intervention (data not shown).

### Adverse events and dropouts

The participants did not report any adverse event during the study period. One participant from each group did not continue the intervention. Thirty-eight participants completed the study. At the end of the intervention, physical performances, changes in arm circumference and the safety parameters were evaluated in nineteen participants from each group. Among the completers, one participant in the placebo and three in GMCT group declined to join in DEXA scan. Hence, we present a comparative analysis of the change from baseline in body composition between GMCT (*n* = 16) and placebo (*n* = 18).

## Discussion

Herbs are popular nutritional supplements and are being considered as ergogenic aids. Botanicals improve exercise performance and endurance by modifying various physiological and psychological factors such as energy metabolism, protein synthesis, androgenic action, adaptability, etc. [2, 4, 29]. However, there are growing concerns over possible side effects of long-term consumption of many plants used in various supplements [29]. Hence, we sought to develop an herbal blend using extracts from plants with a long history of human consumption as food or condiment to ensure the product’s safety.

The present study was conducted to evaluate the hypothesis that endogenous nitric oxide boosting plant products might improve physical performance and endurance of exercise. In the in vitro screening phase, a composition containing aqueous-ethanol extracts of *Garcinia mangostana* fruit rind and *Cinnamomum tamala* leaf at 1:2 (GMCT) showed enhanced nitric oxide production in primary human vascular endothelial cells via endothelial nitric oxide synthase (eNOS) activation (data not shown, to be published elsewhere). Nitric oxide is a potent vasodilator. It regulates exercise-induced vasodilation, which is crucial for maintaining an enhanced supply of oxygen to the working muscle cells; thus can increase physical performance [30].

In addition, our unpublished in vitro observations also indicate that GMCT strongly scavenges reactive oxygen species (ROS) and inhibits NADPH oxidase activity in human neutrophils. Exercising muscles during intense work-outs generate an enormous amount of reactive oxygen and nitrogen species, in majority through the mitochondrial respiratory pathway. Excessive levels of free radicals in the body lead to mitochondrial dysfunction, chromosomal damage, inflammation and cell death [31]. Therefore, it is proposed that a balance between free radical production and their neutralization is crucial for maintaining muscle homeostasis and improving performance [31, 32]. Accordingly, based on the in vitro activities of GMCT, we hypothesized that GMCT might enhance physical performance, strength, and endurance of exercise.

In a preclinical proof of concept study, we were able to show in mice that GMCT increased exercise performances, tolerance, and muscle strength in the experimental animals. Gastrocnemius muscle fibers were thicker in the GMCT supplemented animals, in comparison with those animals which did not receive the supplementation, but the difference was not significant. We strongly believe that a more extended duration study and a larger sample size, we would have been able to detect a significant improvement in muscle fiber size. An increase in the cross-sectional area of muscle fibers is indicative of muscle hypertrophy [33], which is essential for gaining muscle strength [33, 34]. Our preclinical observations also suggested that GMCT might help to increase muscle mass.

We observed an increase in arm circumference of the GMCT supplemented participants in the current clinical trial. In this context, the positive modulatory effect of GMCT in combination with the resistence exercise on the subjects’ free testosterone levels is interesting. Testosterone is an anabolic hormone. It increases muscle mass, strength, and stamina [35–37]. Earlier, Balunas et al. demonstrated that the xanthones from *G mangostana* rind including, α- and γ- mangostins inhibited aromatase activity in cell-free and in-cell models [38].

We theorized that the xanthones in the herbal blend might influence increasing free testosterone concentration via reduced metabolic conversion into estrogens in GMCT supplemented participants. At post-intervention, although the GMCT group showed significant improvement in free testosterone level, the improvement was not significantly different from placebo. Also, GMCT supported increases in lean body mass of the participants. These observations explain that the improved muscle performances and increased lean body mass in GMCT supplemented participants do not depend on increases of testosterone level. We assume that the anabolic activities of GMCT might be due to (i) the ability to increase adaptation to resistance training through an enhanced mitochondrial function, and (ii) by increasing protein synthesis at the ribosomal level via up-regulating mTOR signaling. In future studies, we intend to explore the mechanisms of anabolic actions of GMCT at the molecular level. Overall, in agreement with our hypothesis and preclinical findings, the current trial demonstrates that GMCT increased adaptational response including increases in muscle strength and size, as well as improvements in the endurance of exercise in the resistance-trained participants. It is worth noting that the participants experienced benefit from GMCT supplement as early as 14 days in the form of increased strength and endurance.

Our unpublished observations revealed that *G. mangostana* extract reduced fat gain and accelerated fat breakdown in vitro adipocyte models of 3T3-L1 cells. *G. mangostana* extract inhibited PPARγ dependent adipocyte differentiation process and increased intracellular fat breakdown via AMPK activation in 3T3-L1 mouse adipocytes. These modifications in conjunction with the previously discussed increases in free testosterone may explain the observed beneficial effects of GMCT supplementation on the subjects’ body composition.

Based on the available research findings on safety, tolerability and historical documents on traditional usage of *G. mangostana* rind and *C. tamala* leaf, we did not anticipate any safety concern of GMCT. The observations from a broad spectrum of safety studies including a 28-day sub-acute safety study in rats and genotoxicity studies confirmed the safety of this herbal blend (data to be published elsewhere). Furthermore, the present investigation shows no significant alterations in the parameters of hematology or serum biochemistry, or urine analysis of the GMCT supplemented subjects. Together, these confirm that the herbal formulation GMCT is safe and tolerable for oral consumption in human.

One limitation of the study is the lack of quantitative information on the food intake of the participants during the investigation. We aimed to evaluate the ergogenic effect of the herbal composition in combination with a resistance training protocol in the subjects without altering their regular food habits. Although the participants claimed to have adhered to the instructions, the lack of information on daily intake of calorie or protein limits us to evaluate whether these factors influenced the effect of GMCT supplementation. Therefore, we recommend a dietary analysis of calorie and protein consumption by the subjects in future studies. Another limitation is that we lost some data for analysis either from those subjects who could not attend the DEXA scan or due to technical issues in collecting some serum samples for testosterone analysis. Despite these dropouts, we observed a substantial effect in GMCT supplemented group in comparison with the placebo. However, we firmly believe that the comparative analyses would have been better if we could have included the data that we lost. Future studies in larger a sample size with better measure can address these issues.

## Conclusion

In summary, the present clinical trial demonstrates that GMCT supplementation in combination with resistance training is effective in promoting muscle strength, growth and in improving endurance performance in resistance-trained young males. This study also indicates that the participants have tolerated well the GMCT supplementation, and they have substantial benefit in improving lean body mass in conjunction with a resistance training program. Future studies should be conducted to explore the underlying molecular mechanisms responsible for the anabolic activities of GMCT. Further, it would be interesting to evaluate whether GMCT supplementation can improve muscle functions and physical performances in aging males and untrained young men as well.

## Abbreviations

1-RM: One-Repetition Maximum
ALT: Alanine Aminotransferase
AMPK: 5′ adenosine monophosphate-activated protein kinase
ANCOVA: Analysis of Covariance
AST: Aspartate Aminotransferase
DEXA: Dual-Energy X-Ray Absorptiometry
EIA: Enzyme Immunoassay
eNOS: endothelial Nitric Oxide Synthase
ESR: Erythrocyte Sedimentation Rate
FST: Forced Swim Test
HMB: β-Hydroxy β-Methylbutyrate
NADPH Oxidase: Nicotinamide Adenine Dinucleotide Phosphate Oxidase
OECD: The Organization for Economic Co-operation and Development
PPARγ: Peroxisome proliferator-activated receptor gamma
